# Remote Passive Sensing of Older Adults’ Activities and Function: User-Centered Design Considerations for Behavioral Interventions Conducted in the Home Setting

**DOI:** 10.2196/54709

**Published:** 2024-10-18

**Authors:** Lyndsey M Miller, Jeffrey Kaye, Allison Lindauer, Wan-Tai M Au-Yeung, Nathaniel K Rodrigues, Sara J Czaja

**Affiliations:** 1 Oregon Center for Aging and Technology Oregon Health and Science University Portland, OR United States; 2 School of Nursing Oregon Health and Science University Portland, OR United States; 3 Department of Neurology Oregon Health and Science University Portland, OR United States; 4 Center on Aging and Behavioral Research Division of Geriatrics and Palliative Medicine Weill Cornell Medicine New York, NY United States

**Keywords:** user-centered design, remote passive sensing, remote monitoring, behavioral interventions, caregiving, dementia, Alzheimer, monitoring, gerontology, geriatrics, older adult, aging, usability, acceptability, trust, behavioral

## Abstract

Behavioral intervention studies often lack sufficiently sensitive and frequent measurements to observe an effect. Remote passive sensing offers a highly sensitive, continuous, and ecologically valid method of assessment that increases the ability to detect changes in the daily activities and function of those being monitored. To be most effectively deployed in research studies, applications of remote assessment technology must be designed with the end user in mind. User-centered design (UCD) is especially important in clinical trials where the needs and characteristics of participants and research staff need to be uniquely considered to ensure the feasibility and acceptability of the study. This paper describes UCD issues in remote passive sensing that commonly arise among older adult participants—including those living with dementia—as well as any strategies that were taken to overcome them. Using exemplars from the National Institute on Aging–funded Roybal Center ORCASTRAIT (Oregon Roybal Center for Care Support Translational Research Advantaged by Integrating Technology), as well as other experimental and observational research studies conducted in community settings, this paper brings together our collective experiences with studies using remote passive sensing technology that incorporate a UCD design approach. Although passive sensing eliminates some common UCD issues that arise with higher-touch technology, issues, such as usability, trust, and aesthetic acceptability, still need to be addressed for behavioral interventions using passive sensing technology to be potent and implementable.

## Introduction

As digital health technologies continue to proliferate in both research and clinical care, the uptake of remote passive (or ambient) sensing for noninteractive digital data collection represents a unique opportunity to detect meaningful patterns in older adults’ daily activity over longer periods of time without requiring interaction from participants. Subtle decline in everyday function occurs as part of the normal aging process and as a symptom of pathologies commonly found among older adults, such as Alzheimer disease and related disorders. Likewise, stress from caregiving for older adults with chronic health conditions impacts everyday routines and behaviors such as sleep and physical activity. Detecting and differentiating these subtle changes is ideally pursued in the home setting, where older adults perform their regular daily routines and demonstrate their functional capacity in ecologically valid ways. Remote digital assessment of older adults’ daily activity holds promise for detecting early signs of Alzheimer disease [1]; for intervening to prevent adverse health outcomes, such as falls and the need for emergency care [2-4]; and for understanding the effects of interventions designed to support daily life or caregiving activities [5-8]. Ultimately, remote digital assessment may contribute to helping older adults live longer independently; however, in order to do, so the technology must be designed with the user in mind—in a way that is minimally obtrusive and maximally acceptable to older adults at risk for decline and, in many cases, their family members.

Passive sensing occurs when sensors are embedded in the environment and require no active input from the study participants. The goal is to remotely assess daily function and other regular activities with minimal disturbance to participants’ lives and to increase the ecological validity of assessments by moving the observations from the clinic into the home setting. Examples of passive sensors include bed pressure mats; physical environmental sensors, such as thermostats; or motion sensors on the wall. Contrarily, active sensing requires study participants to actively input data or to perform a task through a digital interface. For example, a mobile app may require users to enter physical activity goals and turn on an activity tracker during exercise. Even wearable sensors, such as actigraphy watches, require some user interaction. Generally, passive sensing is more suitable for long-term monitoring as it requires minimal input from users. Despite the benefits of passive sensing to gain more detailed and unobtrusive insights into participants’ daily lives, issues in the design of passive sensing systems can still interfere with user experience and long-term willingness to participate in such research studies.

User-centered design (UCD) is a design philosophy focused on understanding the users, tasks, and environments [9]. It is also an approach grounded in the characteristics of the individuals who use the innovation [10] and a process that iteratively tests usability and the user experience (ISO 2010). General principles, or heuristics, of UCD, may be overlooked in passive sensing, although this would be an oversight since many of the heuristics are applicable (eg, error prevention and aesthetics) [11]. Researchers who incorporate remote assessment into their studies may be motivated by a variety of reasons to focus on UCD, for example, optimization of enrollment outcomes (eg, faster recruitment) and minimization of attrition. In general, the more passive the technology, the less design of the user interface is required. Yet, UCD issues still exist in noninteractive passive sensing technology and should be considered early and often in order to conduct research that is acceptable or even appealing to participants, thereby improving data quality and retention. The objective of this paper is to provide an overview of UCD issues that pertain to remote passive sensing and to give examples from research studies that use passive sensing.

## Users and UCD Issues in Passive Sensing

### Users

The UCD process begins with identifying end users and understanding their characteristics, abilities, needs, and preferences [9]. Although the primary users of remote assessment technology are the participants who generate the digital activity data by living in the midst of these sensors, secondary users include anyone who installs the sensors and maintains their integrity, and those who access the remote sensor data streams to analyze activity or health outcomes. Depending on the type of research study, secondary users may include clinicians, family members, or other individuals who are involved in the maintenance of, or interaction with, the sensors or the data derived from them.

### UCD Issues

#### Trust

The willingness of users (eg, research participants, family members, and clinicians) to accept remote sensor platforms or other automated systems as a means of detecting meaningful changes in function and daily life is determined in large part by trust [12]. Ideally, the users’ perceptions of the performance and functionality of the remote digital activity monitoring system will match the actual performance and functionality [13]. Yet, “undertrust” of the system occurs at times when users infer nonexistent capabilities, especially those that may invade their privacy. For example, users may worry that passive infrared (PIR) sensors placed in the bathroom are “hearing or seeing” their activities, even though there are no audio or video recording capabilities. Lack of trust is not always unfounded though and may occur when users perceive or experience high error rates (eg, false alarms) or an unreliable system. In the case of remote passive sensing, the unreliability of the system would in most cases only be detected by those users with access to the system’s operational status or the data flowing from the sensors.

#### Error Recognition and Recovery

The ability to recognize errors and the ease of recovering from errors are central to UCD. In a remote passive sensing platform, most participants would not be expected to recognize or infer errors in the transmission of their data unless a sensor is visibly broken or out of place. Participants who are particularly proactive or curious about the functionality of the remote passive sensing platform may become concerned or frustrated by their inability to see the operational status or detect errors (ie, the system is a “black box” to them). In general, study teams working with remote passive sensing would need to reassure participants that they will be in touch if any issues are detected.

When PIR motion sensors are used on the walls or ceilings of a participant’s home, participants will notice when a sensor has fallen on the floor. Recovering from this error can be as simple as sticking the sensor back on the wall in the approximate place where it belonged. However, some studies use a line of motion sensors on the ceiling to detect gait speed. These sensors must be placed more precisely at a certain distance from each other in order to detect gait speed accurately, and so, in this case, error recovery would depend upon the participant reporting the fallen sensor to a technician, who would then come and restore it to the ceiling. With wearable sensors that have a long-term battery life and do not need to be removed from the wrist, “errors” of omission may occur when the participant chooses not to wear it at certain times. These types of errors would likely not be reported by the participants, and error recovery would only occur when secondary users (eg, technicians or other members of the study team) recognized the lapse in data and contacted the primary user to diagnose the error. Lapses in data can occur with any sensor for a variety of reasons, thus the remote passive sensing system must be monitored frequently by the secondary users for data quality errors.

#### Overtrust and Therapeutic Misconception

There is also the potential for users to “overtrust” the purpose of the remote monitoring system (ie, misconstrue the purpose or capability of the system and assume nonexistent benefit). The issue of overtrust typically is discussed in the design of robotics and automated driving systems [14]; however, there are parallels in remote passive sensing as well. With sensors on the walls and doors, some participants in remote monitoring studies could mistake the functionality of sensors to be a home security system that will keep them safe. Older adult participants who wear a sensor may mistake the capability of the system to include a falls alert mechanism. These misconstrued benefits create an ethical issue of therapeutic misconception since participants may not seek the services that they need or desire if they misconceive the remote monitoring system as already providing those services. Just as it must be addressed in drug trials, the boundaries between clinical care and experimental therapies must be well defined [15]. The limits of a remote monitoring system need to be effectively communicated to users to avoid overtrust and therapeutic misconceptions.

#### Aesthetic Acceptability

Aesthetic and minimalistic design are also an important usability heuristic. Although the beauty of the design improves the user experience, only essential information should be presented in the user interface for maximum function [11]. Yet, the primary goal of the design of a passive sensing system is inconspicuousness. Thus, the heuristic must be modified to prioritize unobtrusive design, which allows participants to have minimal contact with the sensors and continue with their daily lives without changing their routines. To accomplish this goal, it is necessary to use sensors that are small in size, have a neutral color, and lack any disturbances from the technology such as lights or beeping that could disturb a participant in their home and make the sensors more obtrusive. For wearable sensors that often appear to be an accessory (eg, actigraphy watches), the initial impression of aesthetic appeal and comfort is important to older adult participants’ attitudes toward using this type of sensor over time [16]. There also should be, as far as possible, minimal maintenance requirements, such as a frequent need to recharge or replace batteries.

#### Data Disaggregation in Multiperson Homes

A UCD issue that pertains to the secondary users who work with the data from remote passive sensing is the inclusion of features that allow the disaggregation of data from homes that include more than one resident. Certain data collected in multiperson homes can be difficult to attribute to a specific person within the home. For example, while the PIR sensors can detect motion within each living space, they do not record pictures or videos, so it is difficult to know whose motion has triggered the sensors. Innovative algorithms are needed to analyze these PIR sensor data to extract meaningful features that would describe the home as one unit. Alternatively, a redesign of the system using more advanced technology that tracked each individual distinctly within the home could be used; however, these improvements for secondary users (those working with the data) would likely detract from the trust of the primary users (the research participants).

Other issues include the framework for data sampling, such as frequency and time, and data management issues, such as approaches for reducing data into meaningful units. The organization and presentation of the data to secondary users must be done in a way that the data are usable and trusted. For example, errors, such as too many false alarms, may result in an undertrust or “boy who cried wolf phenomenon.” Secondary users also need to be trained in the use and meaning of the data.

## Strategies for Addressing UCD Issues

### Overview

In general, UCD strategies aim to make technology more appealing to encourage uptake by users. Paradoxically, UCD strategies for passive sensing generally aim to decrease the need for the user’s interaction with the system and instead to make it as unobtrusive and as “low-touch” as possible. The remote passive sensing issues described in this paper largely concern the assumptions that users make about the sensors, rather than their interaction with them. Thus, the strategies that we have used to mitigate these issues among participants in our research groups must address the users understanding and knowledge about the purpose and function of passive sensing. In research studies, this process begins with informing and educating participants about the system prior to installation and then continues during beta testing and use in research studies.

### Informed Consent

At the National Institute on Aging–funded Roybal Center ORCASTRAIT (Oregon Roybal Center for Care Support Translational Research Advantaged by Integrating Technology), education about the study technologies begins during recruitment and continues throughout study participation. The language used in recruitment materials and during informed consent sets the tone and expectations for participants. Consent forms contain plain language about the function of remote sensing, such as “Please note: the study technologies are not a security system. There is no video, photograph, or audio recording. The technology will not detect a break-in, a fall, or other emergencies. We will not be able to notify anyone in the event of an emergency.”

### Participant Education

**Figure 1:**
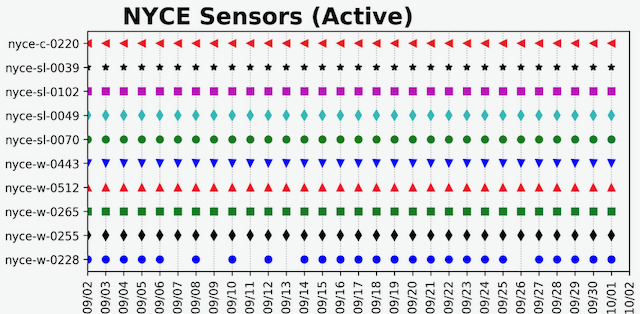
An example of passive infrared motion sensor data shown to participants to mitigate the lack of trust in the anonymous nature of the data.

Educating and re-educating participants continues during studies about the capabilities (or lack thereof) of technology. One way that the ORCASTRAIT teams achieve this is by showing participants examples of their data to illustrate the deidentified nature of the data as it appears to the research team (Figure 1). While showing participants the data depicted in Figure 1, an ORCASTRAIT technician will describe it as follows: “The presence of a shape on the graph indicates that the sensor was activated on that day. This is the 30,000 feet view of the data, zoomed out, giving us an overall picture of the status of the sensors in the homes.”

Users bring their preconceived notions of technology, though, and often retain them even after the study team’s educational efforts. Clearly, the fit between the participant’s goals and the goals of the research will not always be possible and should be taken into account. Yet, a concerted effort must be applied to ensure that we address participants’ concerns and misconceptions when possible, thereby improving readiness. These efforts must be robust in order to include the most representative sample possible since participants from diverse backgrounds vary in terms of digital literacy or misconceptions about remote sensing technology.

## Examples From Intervention Studies

### Overview

UCD principles are frequently considered in the initial development of digital devices and technology interventions; however, UCD is less often considered during the translation and implementation processes of intervention testing, refinement, and expansion [10]. Mental and behavioral health interventions in particular can benefit from applying UCD principles [17]. Recently, the remote monitoring system was developed by the Oregon Center for Aging and Technology (ORCATECH) and used in 2 pilot studies of behavioral interventions funded by the National Institute on Aging–Roybal Center ORCASTRAIT at Oregon Health and Science University (OHSU). These studies were conducted among persons living with dementia and their care partners to detect changes in daily routines such as sleep, physical activity, and time out of the home: READyR (Remote Assessment and Dynamic Response Intervention to Support Co-Residing Care Dyads in Identifying Dementia-Related Care Needs; ClinicalTrials.gov NCT04542109) and STELLA (Support via Technology: Living and Learning with Advancing Alzheimer’s disease and related dementias; ClinicalTrials.gov NCT04335110).

### The READyR Program

The READyR Program is a behavioral intervention designed to assess and target everyday routines of care dyads (older adults with cognitive impairment and their cohabiting partners) using in-home sensors and videoconference sessions in order to understand future care needs and promote greater well-being [18]. In the pilot study of READyR (OHSU Institutional Review Board #20397), ORCATECH infrastructure and a platform of sensors were deployed to collect objective data from remote passive assessments of daily routines. The technology platform used in the READyR study included PIR sensors that captured home exits and activity within or transitions between rooms (NYCE, wireless PIR sensors using a ZigBee network), an actigraphy watch that detected step count (Withings Activité/Steel Watch), an electronic pillbox that captured medication-taking behavior, (TimerCap, wirelessly transmits data about when and which day of the week the pillbox doors were open or closed), and an under-the-mattress bed mat that captured a variety of sleep metrics (eg, bed exits at night and total sleep time) and physiologic data such as heart rate and respirations (Emfit, ballistocardiography sensor).

One of the goals of the READyR intervention was to better assess the alignment of participants’ daily routines with their care values such as autonomy and safety. To help participants visualize their daily activity data in intervention sessions, a tailored web-based tool was created. Data specific to each participant’s home were extracted from the ORCATECH central server. These data were processed and analyzed to generate plots and figures of activity related to participants’ daily routines, which were incorporated into an HTML template to create a static web page that could be displayed for participants.

User experiences with having passive sensors in the home, and with viewing their daily activity data, were video recorded in intervention sessions and acceptability surveys. Of the sample of 24 participant dyads (half of which had cognitive impairment, and the other half were their spouses) who completed the acceptability survey, 23 participants rated the sensor data that were presented to them in web-based intervention sessions as “very easy” or “easy” to understand, and only 1 participant rated it as difficult. The most common user issue was that when presented with data about their daily routines, over half of the participants expressed doubt that the sensors accurately captured at least one of their routines such as time out of home or sleep disturbances. A few participants gave concrete reasons for their doubt, for example:

You might want to check that the sensors are working properly, because one keeps falling off the wall. We try to put it back up, but it doesn’t stick, so I’m wondering if this is really my activity or if it is a sensor thing.

The ORCATECH platform includes multiple ways of assessing some functions, such as sleep, and participants were more reassured that the data represented their patterns when we could show them 2 sources of data that were in agreement.

The issue of therapeutic misconception arose several times during the READyR pilot study. One participant living with Alzheimer disease was prone to somnambulism, and her partner expressed hope that the sensors could offer a way to detect it and alert them, especially when she opened the front door at night. Another participant with mild cognitive impairment went for long bike rides and did not bring his phone. His spouse expressed disappointment that there was no capability to track his whereabouts with the wearable in case he got lost or hurt. A third participant living with Alzheimer disease had a tendency to fall, and his spouse was concerned that there were no sensors in their garage to detect a fall. In all cases, re-educating the participants about the functionality of the sensors, and the lack thereof, was our main strategy for addressing this UCD issue. In a future iteration and testing of the READyR Program, we plan to produce a sensor guide for families to refer to after installation and examine other ways of providing intermittent information to curb misperceptions.

### The STELLA Study

The STELLA study enrolled 13 care partners and their family members with moderate to late dementia. The study tested an intervention designed to help care partners recognize and address behavioral symptoms of dementia, with the primary aim being to reduce care partner burden (OHSU Institutional Review Board #19306). The STELLA study used bed mats to assess sleep duration and quality (under-the-mattress, ballistocardiography-based sensor, Emfit), PIR wall sensors (NYCE) to assess motion, and wearables to assess step counts (Withings Activité/Steel Watch).

After completing the intervention, 4 STELLA care partners participated in a focus group that addressed their perceptions of STELLA and their experiences with technology. Several technology-related user experience themes emerged from the focus groups: (1) a mismatch between study goals and the participants’ needs; (2) trust of the system, or privacy concerns; and (3) and lack of aesthetic and minimalist design.

Care partners found that their needs were not met with the technology in that they had to manage the interaction between their family member with dementia and the sensors: “I should have put them (the sensors) up higher.” His family member with dementia saw “these little things on the wall, and pulls them off. And I find them in her pocket later.” Two STELLA care partners had difficulty convincing their family members to wear the watch. The technology on the watch did not update to adjust to time changes, also leading to frustration, as this care partner noted:

It’s not dependable. I just hate to wake up on the morning when we have daylight saving time and it’s the wrong time and we’re late for church or whatever.

Some STELLA participants also expressed worries about privacy and the “undertrust” discussed above. They were concerned that the team could monitor their activity while they were in bed:

...I’m exhausted. I’m laying here, you know, it’s like, oh, my gosh, they’re noticing that I’m here. You know, it’s like, do they really need to know that kind of thing? Hmm.

Some of the focus group participants did not like the appearance of the sensors, indicating a lack of aesthetic acceptability:

The monitors are on the walls are really ugly. I mean, we got 30 up in the house and, uh, wow. Uh, it’s it doesn’t look good.

Another participant commented that he was somewhat mystified by the wall sensor, indicating the lack of aesthetic acceptability and minimalist design:

The thing on the wall [the wall sensor] was had mysterious numbers all the time...What does all that mean...?

Despite the study team’s efforts to use unobtrusive technology, the STELLA care partners found the devices far from “passive.” The existence of the sensors in the home, even if passive, led some participants to worry about their privacy, struggle to help their family members use devices, like watches, and grapple with malfunctions:

I’m like, this is crazy. This is for people with behavioral disturbances? I sent it back. I don’t like those watches or the motion detector.

It should be noted that all STELLA participants had experience using technological devices such as phones, computers, and email. They all had consistent support from the ORCASTRAIT team:

The gal [technology specialist] that comes to our house, she’s probably been here more than the mailman.

The STELLA focus group revealed that as we tested this intervention that addressed the care partner burden, it is possible we increased the care partners’ sense of daily hassle with the technology.

Overall, the user experience feedback from focus groups may reflect the fact that family caregivers of older adults with later-stage dementia are a distinct type of user that had not been considered in the original design of the passive sensing platform. The additional needs and preferences of a new set of users should be considered [17], and after identifying those distinct needs, the UCD process involves ideation and exploration of prototypes and testing [9]. In this case, the new user group of family caregivers of persons with later-stage dementia will prompt a redesign of the passive sensing platform before testing again with this user group.

### FITTLE

More commonly accepted passive technologies, such as a long-term wearable device, can also highlight UCD issues in behavior intervention studies. One example is from a recent study evaluating a digitally delivered physical activity and social support intervention among community-dwelling older adults (ClinicalTrials.gov NCT03538158), which included the use of wearables (similar to a wristwatch) to objectively assess activity engagement or step count. The participants ranged in age from 65 to 98 years and some had minimal experience with technology. Although a standard commercially available wearable was selected for the study, an issue that arose was a lack of adherence among the participants with respect to the use of wearable. Some participants were not used to wearing a watch, and others were not accustomed to wearing a watch at bedtime. These aspects of comfort and acceptability resulted in gaps in the data when participants forgot to put on the wearable or chose not to wear it at night (SJ Czaja, 2024, unpublished data). This reinforces the need for reminding study participants about the function of the passive technology, and the importance of adherence with respect to study outcomes.

## Examples From Longitudinal Studies

### Overview

Long-term use of a remote sensing platform is often the intended duration. One of the advantages of a remote assessment system is the ability to monitor daily life in the home setting and detect changes over time. However, new UCD issues arise during long-term monitoring that cannot be taken into account during short-term testing of the system.

### MODERATE

MODERATE is a longitudinal study for which the target population is couples living at their residences, with one having dementia and one being the care partner. MODERATE focuses on identifying digital biomarkers of agitated behaviors exhibited by participants living with dementia and identifying environmental precipitants of such behaviors. Passive motion sensors and contact sensors (NYCE, wireless PIR sensors using a ZigBee network), were installed in participants’ homes to continuously assess in-home activity.

Algorithms developed at ORCATECH extract time spent out of home based on whether there was motion detected between doors opening or closing. In single-resident homes, the metric, time spent out of home, can be relatively easy to interpret. However, in double-resident homes, time spent out of home refers to the time both residents are out of home and that does not include the time when only one of them is out of the home. In addition, in double-resident homes, it is difficult to attribute motion detected in a specific room to a particular person, and innovative algorithms are required to synthesize the motion sensor data. We have also developed an algorithm that extracts the duration of independent life-space activities within the home. This metric refers to the duration when there were activities simultaneously detected in two or more life spaces within the home. Such a metric can be used to infer a basic level of physical function and independence of the participants. Yet, the primary users’ unanticipated activity patterns impact the error rate of these algorithms. Long sedentary periods of sitting on the couch may look identical to an unoccupied room. Exiting and re-entering a room quickly may not be recorded as a room transition. Testing these and other behaviors using scripted trials in the home was necessary to identify and recover from these types of errors in the algorithms.

In MODERATE, we also deployed actigraphy devices, research-grade Actiwatch Spectrum, to be worn by people living with dementia, and Withings Activité/Steel Watch, to be worn by caregivers, to track their daily level of activity. Although we have demonstrated powerful examples of behavioral symptoms being detected by actigraphy devices among persons living with dementia [19], we have also encountered “errors” of the actigraphy devices being lost or damaged. One of the wearables was found in the participant’s clothes dryer. In addition, one of the caregivers’ actigraphs was struck by a person living with dementia during a bout of agitation. As a result, the glass surface broke. We learned that there are distinct UCD issues with deploying digital technology in the homes of people living with later-stage dementia as their cognition and behavioral symptoms worsen. In addition, in our research settings, the Actiwatch Spectrums (activity monitoring watch with solid-state “piezo-electric” accelerometer and color-sensitive photodiodes) only have around 4 weeks of battery life. As a result, we continuously need to swap out devices via mailing which is labor-intensive for both our staff and participants.

### Collaborative Aging Research Using Technology

The Collaborative Aging Research Using Technology (CART) initiative was an interagency (National Institutes of Health and Department of Veterans Affairs) program of research funded to spur wider, evidenced-based use of digital technologies in aging research. CART developed and validated protocols and infrastructure in a longitudinal demonstration study, enrolling 301 participants across 4 diverse cohorts (rural residing residents, older adults living in low-income housing, African American older adults living in Chicago, and Latino older adults living in Miami). The project demonstrated that the system could enable digital health monitoring and intervention delivery that is technology agnostic and extensible to many use cases and research scenarios [20]. The scalable system is being used by multiple research groups in North America and Europe [21]; versions of this platform were used in the READyR and STELLA studies described above. In developing the system, priority was placed on ensuring that the methods were incorporated into the daily lives of diverse populations of older adults. User experience feedback was crucial and remains key to the continuous improvement of the experience and engagement of older populations in research; this is crucial to ensuring long-term adherence to research protocols, reducing missing data and maintaining ecological validity.

To highlight UCD principles further, we note some observations from the CART study. For example, the low-income housing cohort (participants living in US Department of Housing and Urban Development, Section 202 subsidized housing) and the rural-residing veterans cohort were found to have overall excellent daily adherence to wearing a wrist-worn watch (Withings Activité/Steel Watch) that recorded step counts and sleep times, with an overall daily wear compliance of 86.5% [22]. However, it was observed that despite using a device chosen for its typical wrist-watch appearance (ie, higher aesthetic acceptability for a research-device wearable), which required battery replacement only every 8-9 months, and for which there was clear participant agreement at the study entry that the watch was intended to be worn throughout the day and night (except for bathing), the wear compliance at night was found to be lower after a mean of 4.7 (SD 1.8) months of study (69.5%) [20]. When asked about the lower wear compliance at bedtimes, participants relayed that they found they were not comfortable wearing any wrist-worn device every night, even though they thought that this would be feasible for them at the beginning of the study. This experience highlights, as noted here and for the READyR and STELLA intervention studies described above, that user-experience needs to be studied under the actual conditions of use. CART participants using the watch in short-term (2 weeks in duration) device validation pilot studies [23] did not voice this potential longer-term compliance issue.

**Figure 2:**
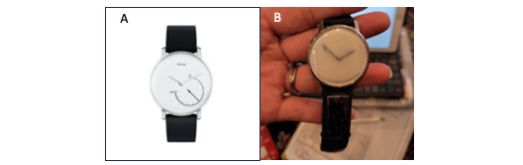
(A) Original watch to be worn in the CART study (Withings Activité). (B) The watch altered by a participant to cover the watch face using tape, with watch hands drawn on so that the participant would not see the watch as a timepiece, as they found the watch not providing accurate time (likely that the battery was wearing down). CART: Collaborative Aging Research Using Technology.

Although the above examples illustrate how participants may choose for understandable reasons to not comply with a protocol because of usability factors and personal life circumstances, we also note that many dedicated participants will invent or devise “workarounds” to enable them to continue in a study. These must be identified during the study so that, for example, if a protocol states that a wearable should be worn all the time (as in our example) or worn on the nondominant wrist, and a participant decides they are more comfortable wearing the device on their other wrist, at variable times, this can add inaccuracies to the data. In some cases, we have discovered that participants devise an alternative that does not affect the data (Figure 2). Participants’ ideas and solutions are welcome, and in fact, the research team may gain valuable knowledge from their relating to that experience. Finding ways to intentionally incorporate the user experience into the design process of remote sensing research can mitigate many UCD challenges.

## Discussion

The proliferation of remote passive sensing in both research and clinical care expands the possibilities for objectively detecting meaningful patterns in function and daily activity over longer periods of time in the home setting. Many of these technologies require minimal or no interaction from participants; however, this does not eliminate UCD issues in remote passive sensing. This paper provides an overview of the most prevalent UCD issues encountered in 5 remote passive sensing studies: 3 behavioral intervention studies (READyR, STELLA, and FITTLE) and 2 longitudinal studies (MODERATE and CART), all conducted with community-dwelling older adult participants living cognitive impairment and their care partners. We describe UCD issues of trust, error recognition and recovery, therapeutic misconception, aesthetic acceptability, and data disaggregation in multiperson homes and the strategies we have used to mitigate these issues broadly, as well as in examples from our research.

The frequent or continuous objective assessment of real-world outcomes of sleep, activity, and objective indicators of stress can greatly increase understanding of the efficacy of behavioral interventions designed to improve these important aspects of well-being. Moreover, taking advantage of passive sensing in order to obtain objective measures for both caregivers and persons living with dementia in the stream of everyday life provides unprecedented opportunities to gain insight into the mechanisms of behavioral interventions. Yet, if participants do not trust or do not accept the use of passive sensing technology in their daily lives, they may change their behavior around it, stop using it when it is inconvenient or uncomfortable (as in the case of the actigraphy watch at night), or withdraw from the study altogether. Clearly, this is not a worthwhile tradeoff if it results in missing data or attrition. In all examples presented in this paper, educating participants was a fundamental strategy to improve the likelihood of acceptability of remote sensing technology; however, it is not a panacea to all UCD issues.

The choice of technologies to be deployed in a study is driven by the specific use case and can also be a tradeoff between desired functionalities for research and usability or acceptability by study participants. Adding more technology to a study for more functionality can in some cases tip the scales toward greater acceptability, as was the case in the READyR study with the participants who wanted more evidence that the daily activity patterns shown in the data reflected their true routines. Having more than 1 sensor stream showing similar data was better in that study. In the STELLA study, participants found the technology to be less acceptable and more burdensome.

## Abbreviations

CART: Collaborative Aging Research Using Technology
OHSU: Oregon Health and Science University
ORCASTRAIT: Oregon Roybal Center for Care Support Translational Research Advantaged by Integrating Technology
ORCATECH: Oregon Center for Aging and Technology
PIR: passive infrared
READyR: Remote Assessment and Dynamic Response
STELLA: Support via Technology: Living and Learning with Advancing Alzheimer’s disease and related dementias
UCD: user-centered design

## References

[1] Hampel H, Au R, Mattke S, van der Flier WM, Aisen P, Apostolova L, Chen C, Cho M, de Santi S, Gao P, Iwata A, Kurzman R, Saykin AJ, Teipel S, Vellas B, Vergallo A, Wang H, Cummings J (2022). Designing the next-generation clinical care pathway for Alzheimer's disease. Nat Aging.

[2] Gaugler JE, Rosebush CA, Zmora R, Albers EA (2022). Outcomes of remote activity monitoring for persons living with dementia over an 18-month period. J Am Geriatr Soc.

[3] Piau A, Mattek N, Crissey R, Beattie Z, Dodge H, Kaye J (2020). When will my patient fall? Sensor-based in-home walking speed identifies future falls in older adults. J Gerontol A Biol Sci Med Sci.

[4] David MCB, Kolanko M, Del Giovane M, Lai H, True J, Beal E, Li LM, Nilforooshan R, Barnaghi P, Malhotra PA, Rostill H, Wingfield D, Wilson D, Daniels S, Sharp DJ, Scott G (2023). Remote monitoring of physiology in people living with dementia: an observational cohort study. JMIR Aging.

[5] Figueiro MG, Pedler D, Plitnick B, Zecena E, Leahy S (2023). Tailored lighting intervention (TLI) for improving sleep-wake cycles in older adults living with dementia. Front Physiol.

[6] Hodgson NA, Gooneratne N, Perez A, Talwar S, Huang L (2021). A timed activity protocol to address sleep-wake disorders in home dwelling persons living with dementia: the healthy patterns clinical trial. BMC Geriatr.

[7] Garshol BF, Ellingsen-Dalskau LH, Pedersen I (2020). Physical activity in people with dementia attending farm-based dementia day care—a comparative actigraphy study. BMC Geriatr.

[8] Wrede C, Braakman-Jansen A, van Gemert-Pijnen L (2023). Understanding acceptance of contactless monitoring technology in home-based dementia care: a cross-sectional survey study among informal caregivers. Front Digit Health.

[9] Czaja SJ, Boot WR, Charness N, Rogers WA (2019). Designing for Older Adults: Principles and Creative Human Factors Approaches.

[10] Dopp AR, Parisi KE, Munson SA, Lyon AR (2019). A glossary of user-centered design strategies for implementation experts. Transl Behav Med.

[11] Nielsen J Ten usability heuristics. Semantic Scholar.

[12] Hoff KA, Bashir M (2015). Trust in automation: integrating empirical evidence on factors that influence trust. Hum Factors.

[13] Kaindl H, Svetinovic D (2019). Avoiding undertrust and overtrust. REFSQ Workshops.

[14] Wagner AR, Borenstein J, Howard A (2018). Overtrust in the robotic age. Commun ACM.

[15] de Bot ST (2019). Raising awareness of therapeutic misconception and optimism around clinical trials in Huntington's disease. J Huntingtons Dis.

[16] Charness N, Best R, Evans J (2016). Supportive home health care technology for older adults: attitudes and implementation. Gerontechnology.

[17] Lyon AR, Koerner K (2016). User-centered design for psychosocial intervention development and implementation. Clin Psychol (New York).

[18] Miller LM, Solomon DN, Whitlatch CJ, Hiatt SO, Wu CY, Reynolds C, Au-Yeung WTM, Kaye J, Steele JS (2022). The remote assessment and dynamic response program: development of an in-home dementia-related care needs assessment to improve well-being. Innov Aging.

[19] Au-Yeung WTM, Miller L, Beattie Z, Dodge HH, Reynolds C, Vahia I, Kaye J (2020). Sensing a problem: proof of concept for characterizing and predicting agitation. Alzheimers Dement (N Y).

[20] Beattie Z, Miller LM, Almirola C, Au-Yeung WTM, Bernard H, Cosgrove KE, Dodge HH, Gamboa CJ, Golonka O, Gothard S, Harbison S, Irish S, Kornfeld J, Lee J, Marcoe J, Mattek N, Quinn C, Reynolds C, Riley T, Rodrigues N, Sharma N, Siqueland M, Thomas N, Truty T, Wall R, Wild K, Wu C, Karlawish J, Silverberg N, Barnes L, Czaja S, Silbert L, Kaye J (2020). The collaborative aging research using technology initiative: an open, sharable, technology-agnostic platform for the research community. Digital Biomarkers.

[21] Thomas NW, Beattie Z, Riley T, Hofer SM, Kaye J (2021). Home-based assessment of cognition and health measures: the collaborative aging research using technology (CART) initiative and international collaborations. IEEE Instrum Meas Mag.

[22] Kaye J, Silbert L, Beattie Z, Mattek N, Wall R, Marcoe J, Sharma N, Riley T, Kornfeld J, Dodge HH (2019). High compliance, continuous long-term home monitoring of sleep activity in diverse populations differentiates lower cognitive functioning older adults. Alzheimers Dementia.

[23] Au-Yeung WTM, Kaye JA, Beattie Z (2020). Step count standardization: validation of step counts from the withings activite using PiezoRxD and wGT3X-BT. Annu Int Conf IEEE Eng Med Biol Soc.

